# Mitochondrial division inhibitor (mdivi-1) decreases oxidative metabolism in cancer

**DOI:** 10.1038/s41416-020-0778-x

**Published:** 2020-03-09

**Authors:** Wenting Dai, Guan Wang, Jason Chwa, Myung Eun Oh, Tharindumala Abeywardana, Yanzhong Yang, Qiong A. Wang, Lei Jiang

**Affiliations:** 1 Department of Molecular and Cellular Endocrinology, Diabetes and Metabolism Research Institute, City of Hope Medical Center, Duarte, CA 91010 USA; 2 Departments of Cancer Genetics and Epigenetics, Beckman Research Institute, City of Hope Medical Center, Duarte, CA 91010 USA; 3 Comprehensive Cancer Center, City of Hope Medical Center, Duarte, CA 91010 USA

**Keywords:** Cancer metabolism, Energy metabolism

## Abstract

**Background:**

Previous studies suggested that mdivi-1 (mitochondrial division inhibitor), a putative inhibitor of dynamin-related protein (DRP1), decreased cancer cell proliferation through inducing mitochondrial fusion and altering oxygen consumption. However, the metabolic reprogramming underlying the DRP1 inhibition is still unclear in cancer cells.

**Methods:**

To better understand the metabolic effect of DRP1 inhibition, [U-^13^C]glucose isotope tracing was employed to assess mdivi-1 effects in several cancer cell lines, DRP1-WT (wild-type) and DRP1-KO (knockout) H460 lung cancer cells and mouse embryonic fibroblasts (MEFs).

**Results:**

Mitochondrial staining confirmed that mdivi-1 treatment and DRP1 deficiency induced mitochondrial fusion. Surprisingly, metabolic isotope tracing found that mdivi-1 decreased mitochondrial oxidative metabolism in the lung cancer cell lines H460, A549 and the colon cancer cell line HCT116. [U-^13^C]glucose tracing studies also showed that the TCA cycle intermediates had significantly lower enrichment in mdivi-1-treated cells. In comparison, DRP1-WT and DRP1-KO H460 cells had similar oxidative metabolism, which was decreased by mdivi-1 treatment. Furthermore, mdivi-1-mediated effects on oxidative metabolism were independent of mitochondrial fusion.

**Conclusions:**

Our data suggest that, in cancer cells, mdivi-1, a putative inhibitor of DRP1, decreases oxidative metabolism to impair cell proliferation.

## Background

Cancer cells utilise elevated cytosolic glycolysis, also known as the Warburg Effect, to supply their rapid proliferation.^1–3^ In comparison, normal cells rely on the mitochondrial tricarboxylic acid (TCA) cycle to meet their energy needs. Glycolysis is a mitochondria-independent metabolic pathway, and numerous cancer studies showed that elevated glycolysis is associated with disrupted mitochondrial structure and impaired mitochondrial function in cancer cells.^4–12^ Still, it is known that mitochondria are intact and fully functional in cancer cells.^13,14^

Mitochondria are the powerhouse of the cell. Unlike other subcellular organelles, mitochondria vary in morphology, from small granules to large threads.^15,16^ In response to nutrient alterations, mitochondria change their morphology, a phenomenon known as mitochondrial dynamics.^17–19^ Mitochondrial dynamics is regulated by several GTPases: dynamin-related protein (DRP1) controls mitochondrial fission,^17,20,21^ optic atrophy 1 regulates the fusion of the inner mitochondrial membrane, while mitofusin 1 and 2 control the fusion of the outer mitochondrial membrane.^16,18^ Increased DRP1 expression was reported to induce fragmented mitochondria in several types of cancer.^4,5,22–24^ In general, fragmented mitochondria have less active oxidative metabolism than the tubular mitochondria, and this has been widely viewed as the cellular foundation for elevated glycolysis in cancer cells.^25^

High expression of DRP1 in cancer may be a potential target for cancer treatment.^4,5,22,24,26–28^ Mitochondrial division inhibitor 1 (mdivi-1) is an efficacious inhibitor of mitochondrial division in yeast and was identified from a chemical screen for compounds that alter mitochondrial morphology.^29^ Not surprisingly, mdivi-1 has been widely used as a mitochondrial fusion inducer.^30^ Studies showed that mdivi-1 treatment altered mitochondrial morphology and cellular oxygen consumption, induced cell death in cancer cells, and limited tumour size in xenograft models.^4,19^ However, a recent study showed that mdivi-1 failed to elongate mitochondria or inhibit recombinant human DRP1 GTPase activity in mouse embryonic fibroblasts (MEFs) and normal neurons, and mdivi-1 reversibly inhibited respiration at mitochondrial complex I to decrease the oxygen consumption rate (OCR).^31,32^ Also, in *Candida albicans*, mdivi-1 phenotypic effects were independent of DNM1 (analogue of DRP1).^32,33^ To examine the metabolic effects of mdivi-1 and DRP1 inhibition in cancer cells, we applied [U-^13^C]glucose isotope tracing to multiple cell lines (H460, A549, and HCT116). Here we show that, in three cancer cell lines, mdivi-1 treatment impaired cell proliferation through limiting oxidative metabolism, rather than glycolysis. Mdivi-1 effects on oxidative metabolism were independent of DRP1 inhibition or mitochondrial fusion induction. Our study indicates that mdivi-1 plays mutually exclusive roles in mitochondrial dynamics and energy metabolism.

## Methods

### Cell culture

H460, H460-DRP1-WT (wild type), and H460-DRP1-KO (knockout) cells were cultured in RPMI medium with 5% foetal bovine serum (FBS; HyClone, CA, USA), 1% penicillin/streptomycin and 4 mM L-glutamine. A549 and HCT116 cells were cultured in Dulbecco’s modified Eagle’s medium (DMEM) with 10% FBS, 1% penicillin/ streptomycin, and 6 mM L-glutamine.

### Mito-tracker staining

Cells were seeded on imaging chambers (Ibidi, WI, USA) overnight before the indicated treatments. Mitochondria were labelled with MitoTracker Green (100 nM; Invitrogen, CA, USA) for 15 min at 37 °C. Complete media (supplemented with 5% FBS, 4 mM L-glutamine, and antibiotics) was used for imaging performed on a Zeiss Imager equipped with a N-Achroplan ×40/0.75 water immersion lens and an AxioCAM MRm digital camera. Images were captured using AxioVision 4.8 and Zeiss Zen software. At least 10 cells per condition were quantified. The *Z*-stack images were processed using the Image J software (NIH, MD, USA).

### Stable isotope tracing

Stable isotope tracing experiments to determine isotope distributions in soluble metabolites were performed as described.^34^ Glucose and glutamine labelled with ^13^C were both purchased from Cambridge Isotope Laboratories (MA, USA). A549 and HCT116 cells’ ^13^C-tracing studies were performed in DMEM medium containing 10% dialysed FBS. Studies of H460 cells were performed in RPMI medium containing 5% dialysed FBS. Subsequently, either the glucose or glutamine pool within these cells was 100% labelled, and the other pool was unlabelled. DMEM lacking glucose and glutamine was prepared from powder (Sigma, MO, USA), then supplemented with 10 mM D[U-^13^C]glucose and 3 mM unlabelled glutamine. RPMI lacking glucose or glutamine was prepared from powder (Sigma), then supplemented with either 10 mM D[U-^13^C]glucose or 3 mM L[U-^13^C]glutamine. Cells were grown in 60-mm dishes until 80% confluent, treated with or without dimethyl sulfoxide (DMSO) and 20 and 50 μM mdivi-1 for 6 h, and then cultured with ^13^C-labelled medium containing the corresponding mdivi-1 treatments for another 2 h. To further examine the effects of time on oxidative metabolism, H460 cells were treated with ^13^C-labelled glucose medium containing DMSO and 20 μM mdivi-1 for 2, 8, and 24 h. To examine the effects of reactive oxygen species (ROS) levels on oxidative glucose metabolism in combination with mdivi-1, we treated H460 cells with DMSO, 20 μM mdivi-1, and 150 µM H_2_O_2_ with or without 10 mM *N*-acetyl-L-cysteine (NAC) in each group and performed the [U-^13^C]glucose tracing for 2 h. To explore the effects of NADH levels on oxidative metabolism, we treated H460 cells with DMSO, 500 µM nicotinamide mononucleotide (NMN), and 100 nM FK866 and performed the [U-^13^C]glucose tracing for 2 h. After treatments, cells were extracted by freeze–thawing three times in 0.9 mL of a cold 1:1 mixture of methanol and water. Macromolecules and debris were removed by centrifugation (12,000 rpm) for 15 min at 4 °C. Subsequently, the supernatants with aqueous metabolites were evaporated, derivatised for 2 h at 42 °C in 50 μL of methoxyamine hydrochloride (Sigma) and 100 μL *N*-tert-butyldimethylsilyl-*N*-methyltrifluoroacetamide (Sigma) for 90 min at 72 °C. Metabolites were analysed using an Agilent 7890B gas chromatograph (Agilent, CA, USA) networked to an Agilent 5977B mass selective detector. Retention times and mass fragmentation signatures of all metabolites were validated using pure standards. To determine the relative metabolite abundance across samples, the area of the total ion current peak for the metabolite of interest was compared to that of the internal standard and normalised for protein content. The mass isotopomer distribution analysis measured the fraction of each metabolite pool that contained every possible number of ^13^C atoms: a metabolite could contain 0, 1, 2, …*n* ^13^C atoms, where *n* = the number of carbons in the metabolite. For each metabolite, an informative fragment ion containing all carbons in the parent molecule was analysed by the MATLAB software (MathWorks, CA, USA). The abundance of all mass isotopomers was integrated from *m* + 0 to *m* + *n*, where *m* = the mass of the fragment ion without any ^13^C. The abundance of each mass isotopomer was then corrected mathematically to account for natural abundance isotopes and finally converted into a percentage of the total pool.

### CRISPR/Cas9 DRP1 or isocitrate hydrogenase (IDH) knockout H460 cells

DRP1 and IDH1- and IDH3-deficient H460 cell lines were generated using the CRISPR/Cas9 system.^35,36^ WT clones were selected from both the control vector (Vector) and targeting vector transfections (WT). In order to control the variations among individual clones, four to five clones were pooled together, and different pools for each targeted gene were used for further experiments.

### Cell death assay

H460, A549, and HCT116 cells were cultured in 60-mm dishes. Once 80% confluent, cells were treated with DMSO and 20 and 50 μM mdivi-1 for 24 h and then collected. Collected cells were stained by 0.4% trypan blue (Gibco, Thermo, MA, USA) in a 1:1 ratio on a haemocytometer and incubated for 3 min. Subsequently, the live (unstained) and dead (stained) cells were counted. Each treatment was replicated three times.

### Cell apoptosis assay

H460, A549, and HCT116 were cultured in 60-mm dishes. Once 80% confluent, cells were treated with DMSO and 20 and 50 μM mdivi-1 for 24 h and then collected for staining with the Annexin V Apoptosis Detection Kit (eBioscience, Thermo, MA, USA) according to the manufacturer’s instructions. Briefly, cells were washed with phosphate-buffered saline and binding buffer, followed by incubation with 5 μL allophycocyanin-conjugated Annexin V per 5 × 10^5^ cells for 15 min at room temperature. After washing with binding buffer, cells were stained with 5 μL propidium iodide and analysed by flow cytometry (Accuri C6, BD, NJ, USA) immediately. Each analysis was replicated three times.

### Western blot analyses

The protein concentrations of cell lysate were determined by the BCA protein assay using bovine serum albumin as a standard (Thermo, MA, USA). An equal amount of protein (10 μg) per sample was separated on 10% sodium dodecyl sulfate polyacrylamide gels and then transferred onto polyvinylidene difluoride membranes (Millipore, MA, USA). The membranes were blocked with 4% non-fat milk in TBST buffer at room temperature for 2 h, followed by incubation at 4 °C overnight with primary DRP1 (Cell Signaling Techchnology, MA, USA; dilution 1:2000), IDH1 (Cell Signaling Techchnology; dilution 1:2000), IDH3A (Cell Signaling Techchnology; dilution 1:2000), and β-actin (Sigma; dilution 1:4000) antibodies. After washing with TBST three times, the membranes were incubated with secondary goat-anti-rabbit IgG (Bio-Rad Laboratories, CA, USA; dilution 1:3000) or goat-anti-mouse IgG (Bio-Rad, dilution 1:3000) conjugated with horseradish peroxidase in 4% non-fat milk for 2 h at 37 °C. Membranes were then washed with TBST three times and incubated with enhanced chemiluminescence western blotting substrate (Thermo), followed by visualisation using an Amersham Imager 680 system (GE Healthcare, MA, USA). The level of β-actin was used as an internal control.

### IDH1 activity assay

The IDH1 activity assay was adapted from the published protocols.^37,38^ Reactions for each assay contained cell lysate (4 μg), 50 mM Tris, pH 7.5, 100 mM NaCl, 5 mM MgCl_2_, 0.5 mM NADP+, and 0.5 mM isocitrate. The change in absorbance at 340 nm due to the reduction of NADP+ was measured using a BioTek synergy H4 hybrid multimode microplate reader (Thermo).

### Statistical analysis

Significance in metabolite enrichment (and relative levels of metabolites), percentage of dead cells (and relative cell growth rate), and IDH1 enzyme activity in H460, A549, and HCT116 cell lines were determined by using one-way analysis of variance (ANOVA) with Tukey’s test for multiple comparisons among the different mdivi-1 treatment groups. For the metabolic data of H460 cells treated with 20 μM mdivi-1, metabolite enrichment was compared to DMSO controls using the paired Student’s *t* test. For the DRP1-KO (H460 and MEF with no mdivi-1 treatment) metabolic data, metabolite enrichment was compared to their paired DRP1-WT counterpart using the paired Student’s *t* test. For the metabolic data of DRP1-KO (H460 and MEF) cells treated by mdivi-1, the *m* + 2 of α-ketoglutarate (α-KG) enrichment was analysed by two-way ANOVA. A *p* value <0.05 was considered significant. All statistical tests were two tailed. All statistical tests were calculated using the Prism software (GraphPad, CA, USA).

## Results

### Mdivi-1 inhibits oxidative metabolism in H460 cells

**Fig. 1:**
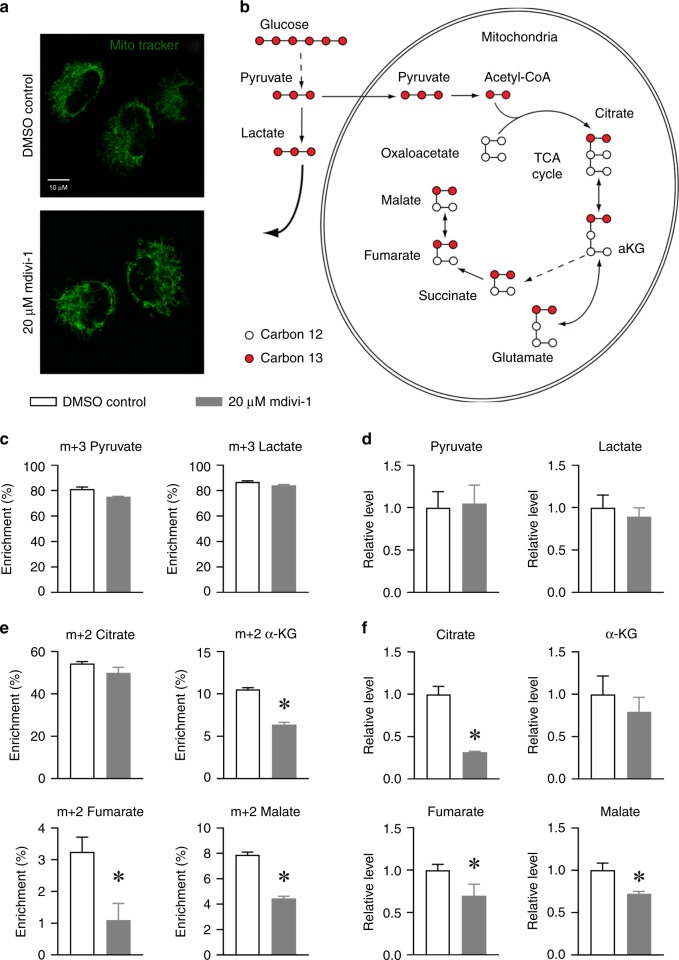
Acute mdivi-1 treatment inhibits oxidative metabolism in H460 cells. **a** MitoTracker staining of H460 lung cancer cells treated with DMSO (control) and 20 μM mdivi-1 for 2 h. Scale bar: 10 μm. **b** An illustration of the first-round metabolic enrichment of glycolytic and TCA cycle intermediates in [U-^13^C]glucose-cultured cells. The empty dots represent the natural carbon 12, and the solid dots represent the carbon 13 isotope. **c**–**f** H460 cells were treated with DMSO (control) and 20 μM mdivi-1 in medium containing [U-^13^C]glucose for 2 h. **c** The metabolic enrichment of glycolytic intermediates is shown as *m* + 3 pyruvate and lactate. **d** The relative levels of pyruvate and lactate. **e** The metabolic enrichment of TCA cycle intermediates is shown as *m* + 2 citrate, α-ketoglutarate (α-KG), fumarate, and malate. **f** The relative levels of citrate, α-KG, fumarate, and malate. Data are represented as mean ± S.D (*n* = 3). The data were analysed by Student’s *t* test, and the significant level was set as *p < 0.05, compared to DMSO control.

Mdivi-1 promotes mitochondrial fusion. H460 lung cancer cells treated with 20 µM mdivi-1 for 8 h showed increased mitochondrial fusion (Fig. 1a). Upon uptake, glucose is metabolised through glycolysis to pyruvate, which enters mitochondria to be oxidised through the TCA cycle. To examine the role of mdivi-1 on glucose metabolism, H460 lung cancer cells were treated with mdivi-1 and [U-^13^C]glucose tracing assessed at 2, 8, and 24 h. The first round metabolic enrichment of glycolysis and TCA cycle intermediates in [U-^13^C]glucose-cultured cells is illustrated in Fig. 1b. Cells treated with 20 µM mdivi-1 treatment did not show a change in enrichment of *m* + 3 (containing three carbons from [U-^13^C]glucose tracer) pyruvate and lactate regardless of the time interval (Fig. 1c and Fig. S1a, b). Similarly, 2 h of mdivi-1 treatment did not change the levels of pyruvate and lactate (Fig. 1d). Upon mitochondrial pyruvate uptake, citrate is the product of first TCA cycle reaction. In H460 cells, treatment with 20 µM mdivi-1 from 2 to 8 h did not change *m* + 2 (containing two carbons from [U-^13^C]glucose tracer) citrate enrichment (Fig. 1e and Fig. S1c). Surprisingly, the levels of other *m* + 2 TCA cycle intermediates (α-KG, fumarate, and malate) were significantly less in the treated cells (Fig. 1e and Fig. S1c). Furthermore, the relative levels of citrate, fumarate, and malate were all reduced in treated H460 cells by 2 h (Fig. 1f). Since attaining a steady state of TCA cycle intermediates requires time, mdivi-1-treated H460 cells were incubated with [U-^13^C]glucose tracer for 24 h. The *m* + 2 enrichment of fumarate and malate were significantly decreased, although there were no changes in the *m* + 2 enrichment of α-KG (Fig. S1d). Conversely, the *m* + 3 and *m* + 4 enrichment of α-KG was significantly less in cells treated with mdivi-1 for 24 h (Fig. S1e). These data indicate that mdivi-1 limits the TCA cycle, without altering glycolysis.

### Mdivi-1 inhibits oxidative metabolism in multiple cancer cell lines

**Fig. 2:**
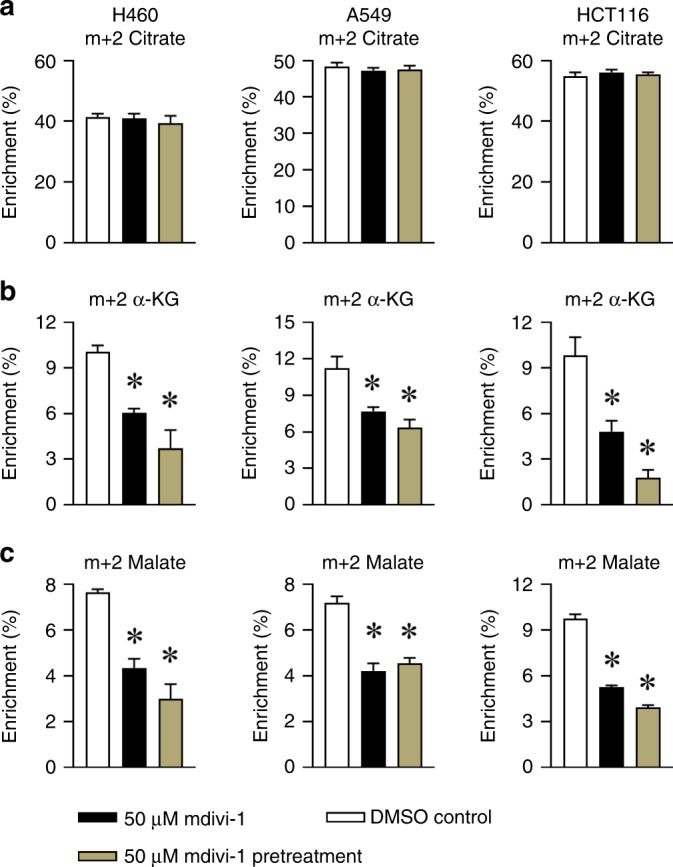
High-concentration (50 μM) mdivi-1 treatment inhibits oxidative metabolism in multiple cancer cell lines. H460, A549, and HCT116 cancer cells were pretreated with or without 50 μM mdivi-1 for 6 h and then cultured in medium containing [U-^13^C]glucose for 2 h. The metabolic enrichment of TCA cycle intermediates is shown as *m* + 2 citrate (**a**), α-ketoglutarate (α-KG) (**b**), and malate (**c**) in all cancer cell lines. Data are represented as mean ± S.D (*n* = 3). The data were analysed by one-way ANOVA followed by Tukey’s multiple comparisons test, and the significant level was set as *p < 0.05, compared to DMSO control.

A recent study showed that acute mdivi-1 treatment decreased oxygen consumption without altering mitochondrial fusion in cultured neurons.^31^ To directly evaluate the role of mdivi-1 on glucose metabolism in cancer cells, multiple cancer cell lines were incubated with [U-^13^C]glucose tracer after pretreatment with mdivi-1 or DMSO for 6 h. An incubation interval of 2 h with [U-^13^C]glucose tracer was selected, since mdivi-1 treatment showed similar effect on the enrichment of TCA cycle intermediates in H460 cells incubated with [U-^13^C]glucose tracer for 2, 8, and 24 h. As several studies used 50 μM mdivi-1 to induce fusion in cancer cells,^29,39,40^ we analysed the metabolic effects of 50 μM mdivi-1 in multiple cancer cell lines, including lung cancer cells (H460 and A549) and colon cancer cells (HCT116). Six-hour pretreatment of cells with 50 μM mdivi-1 did not alter *m* + 2 citrate enrichment (Fig. 2a). In comparison, 6-h pretreatment of 50 μM mdivi-1 significantly reduced *m* + 2 α-KG and malate enrichment in all cell lines (Fig. 2b, c). The decrease in levels of α-KG did not necessarily require mdivi-1 treatment, since cells treated for 2 h demonstrated similar metabolite levels as those treated for 6 h (Fig. 2a). These data suggest that mdivi-1-mediated limitation of oxidative metabolism might not depend on the induction of mitochondrial fusion.

Similar to the H460 cells treated with 20 μM mdivi-1 for 2 h (Fig. 1f), the levels of citrate and malate were decreased by 2 and 6 h of treatment with 50 μM mdivi-1 in all cell lines (Fig. S2 and Table S1). In addition to citrate and malate, only the level of aspartate was decreased following treatment with 50 μM mdivi-1 for 2 h, but none of the other amino acids or glycolytic intermediates showed consistent decreases upon mdivi-1 treatment (Table S1). Since aspartate and oxaloacetate rapidly exchange their carbon backbones,^41^ decreased aspartate levels indicated decreased oxaloacetate levels. More importantly, the level of fumarate was decreased following treatment with 50 μM mdivi-1 for 6 h (Table S1). The decreased levels of TCA cycle intermediates suggest that mdivi-1 treatment decreases oxidative metabolism, without impairing glycolysis or amino acid metabolism.

As high concentrations of mdivi-1 (50 μM) might have unexpected side effects,^30,42^ 20 μM mdivi-1 was tested after 6 h of pretreatment and 2 h of acute treatment in all three cell lines. After 6 h of pretreatment, *m* + 2 citrate levels were not changed by 20 μM mdivi-1 in all cell lines (Fig. S3a). In contrast, *m* + 2 α-KG and malate were significantly decreased by pretreatment with 20 μM mdivi-1 for 6 h in all the cell lines (Fig. S3b, c). Treatment of H460 and HCT116 cells with 20 μM mdivi-1 for 2 h decreased *m* + 2 α-KG levels and also decreased *m* + 2 malate in H460 and A549 cells. Compared to 50 μM, 20 μM mdivi-1 was less effective in decreasing oxidative metabolism. Collectively, the data indicate that high dose (50 μM) mdivi-1 treatment decreases oxidative metabolism in multiple cell lines but that A549 and HCT116 cells are less sensitive to this effect.

### Mdivi-1 treatment decreases glutamine oxidation in H460 cells

**Fig. 3:**
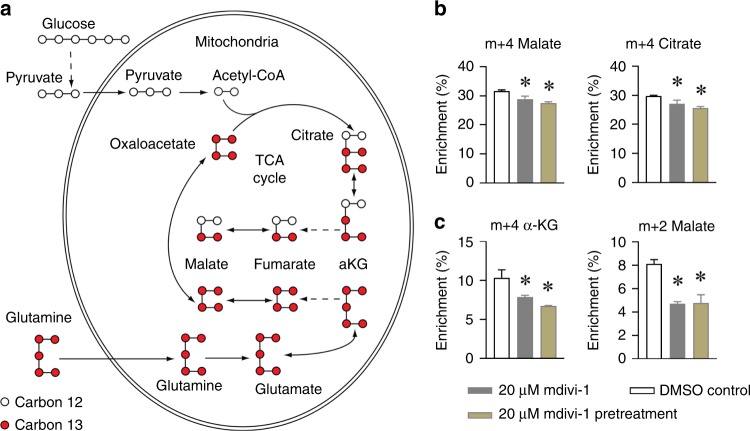
Mdivi-1 treatment decreased glutamine oxidation in H460 cells. **a** An illustration of the metabolic enrichment of TCA cycle intermediates in [U-^13^C]glutamine-cultured cells. The empty dots represent the natural carbon 12, and the solid dots represent the carbon 13 isotope. H460 cells were pretreated with or without 20 μM Mdivi-1 for 6 h and then cultured in medium containing [U-^13^C]glutamine for 2 h. **b** The metabolic enrichment from the first round of oxidative glutamine metabolism are shown as *m* + 4 malate and citrate. **c** The metabolic enrichment from the second round of oxidative glutamine metabolism are shown as *m* + 3 α-ketoglutarate (α-KG) and *m* + 2 malate. Data are represented as mean ± S.D (*n* = 3). The data were analysed by one-way ANOVA followed by Tukey’s multiple comparisons test, and the significant level was set as *p < 0.05, compared to DMSO control.

In addition to glucose, glutamine is another major energy source for cancer cells.^43^ In the [U-^13^C]glutamine tracing assay, glutamine carbons enter the TCA cycle as fully labelled *m* + 5 α-KG (containing all five carbons from [U-^13^C]glutamine tracer). TCA cycle intermediates were labelled as *m* + 4 in the first round of TCA cycle reactions (Fig. 3a). Consistent with previous results (Fig. S3), levels of *m* + 4 malate and citrate were decreased following 20 μM mdivi-1 treatment (Fig. 3b). The final product of the first round of the oxidative glutamine pathway, *m* + 4 citrate, can be further oxidised to *m* + 3 α-KG and *m* + 2 malate through the second round of oxidation (Fig. 3a). Mdivi-1 treatment significantly decreased *m* + 3 α-KG and *m* + 2 malate production in H460 cells (Fig. 3c). These data demonstrate that mdivi-1 treatment decreases oxidative metabolism of glucose and glutamine, two major energy substrates, in H460 lung cancer cells.

### Mdivi-1 inhibits oxidative metabolism in DRP1-deficient H460 cells

**Fig. 4:**
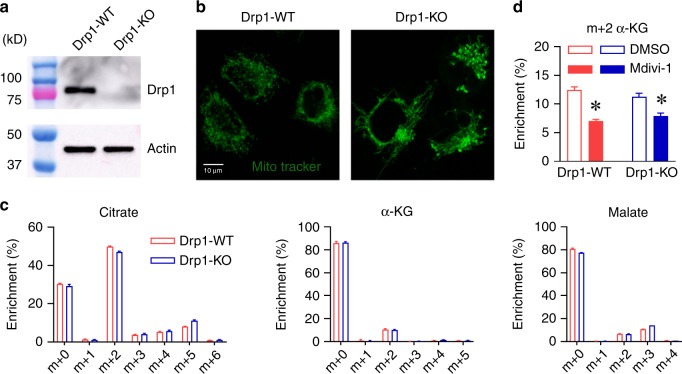
Mdivi-1 inhibits oxidative metabolism in the DRP1-deficient H460 cells. **a** Immunoblotting of DRP1 protein in the Drp1-WT and Drp1-KO H460 cells. **b** MitoTracker staining of Drp1-WT and Drp1-KO H460 cells. Scale bar: 10 μm. **c** Metabolic enrichment of citrate, α-ketoglutarate (α-KG), and malate in Drp1-WT and Drp1-KO H460 cells cultured with [U-^13^C]glucose for 2 h. **d** Metabolic enrichment of *m* + 2 α-KG in cells pretreated with or without 20 μM mdivi-1 for 6 h and cultured in medium containing [U-^13^C]glucose for 2 h. Data are represented as mean ± S.D (*n* = 3). The data in **c** were analysed by Student’s *t* test. The data in **d** were analysed by two-way ANOVA followed by Tukey’s multiple comparisons test, and the significant level was set as *p < 0.05 (20 μM mdivi-1 versus DMSO).

Since mitochondrial fusion is correlated with more active oxidative metabolism, it was surprising that oxidative metabolism was decreased by the mitochondria fission inhibitor agent mdivi-1. Although mdivi-1 is believed to limit DRP1 to induce mitochondrial fusion, a recent study reported a DRP1-independent function of mdivi-1.^31^ To examine the role of DRP1 in mdivi-1 inhibition of oxidative metabolism, we generated DRP1-deficient H460 cells, through CRISPR/Cas9 genome editing.^35^ After the single-cell cloning selection, four to five cell clones were pooled as DRP1-KO and DRP1-WT. Western immunoblot showed the complete deletion of DRP1 protein in DRP1-KO H460 cells (Fig. 4a). Mitochondrial staining confirmed that DRP1-KO H460 cells had extremely high rates of mitochondrial fusion (Fig. 4b), and this was greater than mdivi-1-treated H460 parental cells (Fig. 1a). In [U-^13^C]glucose tracing assays, similar patterns of TCA cycle intermediates were observed in the DRP1-WT and DRP1-KO H460 cells (Fig. 4c). Surprisingly, 20 μM mdivi-1 treatment resulted in a similar lowering of *m* + 2 α-KG levels in DRP1-WT and DRP1-KO H460 cells (Fig. 4d). DRP1-WT and DRP1-KO mouse embryonic fibroblasts (MEFs) also showed similar levels of α-KG and malate, but 50 μM mdivi-1 treatment did not decrease *m* + 2 α-KG levels in either DRP1-WT or DRP1-KO MEFs (Fig. S4). These data suggest that, in H460 lung cancer cells, mdivi-1-mediated decrease in metabolism is independent of DRP1.

### Mdivi-1 induces ROS to inhibit oxidative metabolism in H460 cells

**Fig. 5:**
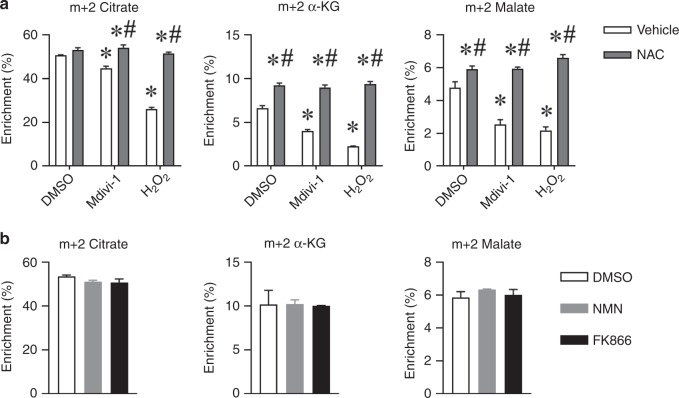
Mdivi-1- and superoxide-mediated inhibition of oxidative metabolism is mitigated by ROS scavenging. **a** H460 cells were treated with DMSO, 20 µM mdivi-1, or 150 µM H_2_O_2_, with or without the co-treatment of 10 mM NAC for 2 h. The metabolic enrichment of TCA cycle intermediates is shown as *m* + 2 citrate, α-KG, and malate. **b** H460 cells were treated with 500 µM NMN or 100 nM FK866 for 2 h. NAC *N*-acetyl-L-cysteine, α-KG α-ketoglutarate, NMN nicotinamide mononucleotide; FK866 is an inhibitor of nicotinamide phosphoribosyltransferase (NAMPT). The metabolic enrichment of TCA cycle intermediates is shown as *m* + 2 citrate, α-KG, and malate. Data are represented as mean ± S.D (*n* = 3). The data in **a** were analysed by two-way ANOVA followed by Tukey’s multiple comparisons test. The significant level was set as *p < 0.05 compared to DMSO control, and ^#^*p* < 0.05 compared to vehicle (NAC versus vehicle). The data in **b** was analysed by one-way ANOVA followed by Tukey’s multiple comparisons test.

Mdivi-1 can induce ROS production in MEFs,^31^ and in the TCA cycle, ROS can inactivate enzymes like succinate dehydrogenase^44^ and alpha-ketoglutarate dehydrogenase.^45,46^ To further examine the role of ROS in mdivi-1-mediated inhibition of oxidative metabolism, we treated H460 cells with H_2_O_2_ (150 µM). Superoxide treatment significantly decreased the levels of *m* + 2 citrate, α-KG, and malate (Fig. 5a). In H_2_O_2_- and mdivi-1-treated H460 cells, these changes were prevented by co-treatment with the ROS scavenger NAC (Fig. 5a), suggesting that mdivi-1 decreased oxidative metabolism through increasing pathologic ROS. Mdivi-1 was reported to reversibly inhibit mitochondrial complex I.^31^ Importantly, complex I inhibition alters mitochondrial NAD+/NADH ratio, which could further regulate the activity of TCA cycle enzymes. To test the role of the NAD+/NADH ratio in mdivi-1-mediated inhibition of oxidative metabolism, we treated H460 cells with NMN (500 µM), to increase the cellular NAD+ level,^47^ and FK866 (100 nM), an inhibitor of nicotinamide phosphoribosyl transferase, to decrease the cellular NAD+ level.^48^ Neither NMN or FK866 treatment altered the levels of *m* + 2 citrate, α-KG and malate in H460 cells (Fig. 5b), suggesting that alteration of NAD+/NADH ratio had no effect on oxidative metabolism in H460 lung cancer cells.

In most mammalian cells, oxidation of citrate to α-KG is primarily catalysed by IDH3, a TCA cycle enzyme.^49,50^ A similar cytosolic oxidative reaction is catalysed by IDH1.^35^ Baseline IDH1 protein levels were higher in H460 compared to A549 and HCT116 cells (Fig. S5a). As H460 cells were more sensitive to mdivi-1 (Fig. 2 and Fig. S3), mdivi-1 might inhibit IDH1 activity to decrease α-KG production. To test this, we performed [U-^13^C]glucose tracing on IDH1-KO and IDH3-KO H460 cells. Both IDH1-KO and IDH3-KO H460 cells showed lower levels of *m* + 2 α-KG, which were further attenuated by mdivi-1 treatment (Fig. S5b). However, in vitro IDH1 enzyme activity was not altered by mdivi-1 (Fig. S5c). In addition, mdivi-1 treatment did not alter the protein expression of IDH1 and IDH3A (Fig. S5d). These data suggest that mdivi-1-mediated effects are linked to pathologic ROS production and altered IDH enzyme activity.

### Mdivi-1 inhibition of oxidative metabolism is independent of mitochondrial fusion

**Fig. 6:**
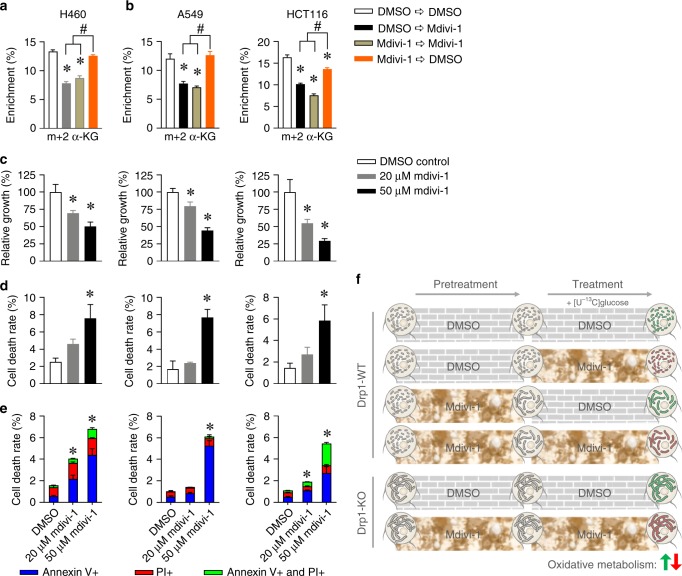
Mdivi-1-mediated inhibition of oxidative metabolism was independent of mitochondrial fusion. **a** The metabolic enrichment of *m* + 2 α-KG in H460 cells first pretreated with or without 20 μM mdivi-1 for 6 h and then cultured in medium containing [U-^13^C]glucose with or without 20 μM mdivi-1 for another 2 h. **b** The metabolic enrichment of *m* + 2 α-KG in A549 and HCT116 cells pretreated with or without 50 μM mdivi-1 for 6 h and then cultured in medium containing [U-^13^C]glucose with or without 50 μM mdivi-1 for another 2 h. **c**–**e** H460, A549, and HCT116 cells were treated with DMSO and 20 and 50 μM mdivi-1 for 24 h, and cell numbers were counted after trypan blue staining. The dead cells were also quantified by Annexin V and PI staining. **c** The relative growth is shown as the number of live cells (trypan blue negative), normalised to DMSO control. **d** The cell death rate is shown as the percentage of trypan blue-positive cells. **e** The dead cells were also quantified by Annexin V and PI staining. **f** Scheme displaying mechanism that mdivi-1-mediated inhibition of oxidative metabolism is independent of DRP1 inhibition or mitochondrial fusion induction. Data are represented as mean ± S.D (*n* = 3). The data in **a**–**d** were analysed by one-way ANOVA followed by Tukey’s multiple comparisons test, and the significant level was set as *p < 0.05, compared to DMSO control, ^#^*p* < 0.05 as indicated in **a**, **b**. The total cell death rate in **e** was analysed by one-way ANOVA followed by Tukey’s multiple comparisons test, and the significant level was set as *p < 0.05, compared to DMSO control.

To further elucidate whether the effects of mdivi-1 involve changes in mitochondrial fusion, we treated H460 cells with 20 μM mdivi-1 for 6 h, removed the medium, added fresh medium lacking mdivi-1, and then performed the [U-^13^C]glucose tracing. Although mdivi-1 treatment induced mitochondrial fusion, the mdivi-1 washout and DMSO control groups showed similar levels of *m* + 2 α-KG, which were significantly higher than the levels of *m* + 2 α-KG in mdivi-1-treated H460 cells (Fig. 6a). The mdivi-1 washout and DMSO control groups also demonstrated similar levels of *m* + 2 α-KG in 50 μM mdivi-1-treated A549 cells, and mdivi-1 washout did not fully reverse mdivi-1-mediated inhibition of oxidative metabolism in HCT116 cells (Fig. 6b). These findings suggest that mdivi-1 inhibition of α-KG production is independent of mdivi-1 effects on mitochondrial fusion. More importantly, treatment with 20 and 50 μM mdivi-1 significantly decreased cancer cell proliferation. Specifically, cells treated with high concentration (50 μM) of mdivi-1 proliferated half as much as DMSO-treated cells (Fig. 6c), and this was associated with increased cell death (Fig. 6d) and apoptosis (Fig. 6e and Fig. S6). Altogether, our data suggested that mdivi-1 treatment impaired cancer cell proliferation through repressing oxidative metabolism, which was independent of DRP1 inhibition or mitochondrial fusion induction.

## Discussion

As the powerhouse of the cell, mitochondria constantly undergo morphologic changes, between large thread (*mitos* in Greek) and small granule (*chondrion* in Greek), as highlighted in the organelle name.^15,16^ Mitochondrial fusion and fission contribute to these morphologic changes and are often dysregulated under pathological conditions, including cancers.^18,19,25,28,51^ Most studies of cancer-related mitochondrial dynamics focus on their function in apoptosis,^52–54^ and elevated mitochondrial fission has been linked to impaired oxygen consumption in cancer cells.^18,55–57^ However, it is not clear whether mitochondrial dynamics regulate metabolic alterations in cancer.

The GTPase DRP1 promotes mitochondrial fission, and high DRP1 expression is reported in several cancer types.^22,24,26,28^ Reducing DRP1 activity is considered as a potential therapeutic approach for cancer. Mdivi-1 (a widely accepted DRP1 inhibitor) induced cancer cell death and decreased tumour size in xenograft models.^4,19^ Mdivi-1 increased OCR in non-cancer cells like mouse neuroblastoma^58^ but deceased OCR in human ductus arteriosus smooth muscle cells.^59^ Similar to these findings, studies showed that mdivi-1 increased OCR in some cancer cells^60,61^ but decreased OCR in other cancer cells.^25,62^ To better understand the regulatory role of DRP1 in cancer metabolism, we used [U-^13^C]glucose tracing to directly reveal the intracellular glucose metabolism in DRP1-deficient H460 lung cancer cells and in multiple mdivi-1-treated cancer cell lines. Our data show that mdivi-1 treatment decreased oxidative metabolism in three cancer cell lines, and DRP1 deficiency did not alter glucose metabolism in H460 lung cancer cells (Fig. 6f).

Further, [U-^13^C]glucose tracing studies showed that acute and chronic mdivi-1 treatment decreased glucose metabolism in multiple cancer cells. This was independent of induced mitochondrial fusion, as control cells and mdivi-1-treated cells had similar oxidative metabolism after the washout of mdivi-1. This fusion-independent role of mdivi-1 is consistent with a recent study which demonstrated that mdivi-1 reversibly inhibited mitochondrial complex I to reduce OCR without altering mitochondrial fusion.^31^ The same study reported that DRP1-WT and DRP1-KO MEFs had similar OCR, which was repressed by mdivi-1 regardless of cell type.^31^ Similarly, we found that levels of TCA cycle intermediates in DRP1-KO MEFs and H460 lung cancer cells were similar to the levels in DRP1-WT cells. Also, mdivi-1 treatment decreased oxidative metabolism to a comparable degree in DRP1-WT and DRP1-KO H460 lung cancer cells, although 50 μM mdivi-1 treatment did not alter glucose metabolism in MEFs. Together these data support the hypothesis that mdivi-1 decreases oxidative metabolism independent of increased mitochondrial fusion or DRP1 inhibition.

In addition, mdivi-1 treatment resulted in significant decreases in the levels of enriched α-KG, suggesting that mdivi-1 might impair IDHs activity. Mdivi-1 treatment did not alter the expression levels of IDH1 and IDH3 or impair the enzymatic activity of purified IDH1. The activity of TCA cycle enzymes, including IDHs, is tightly regulated by redox homoeostasis, and mdivi-1 reversibly inhibited respiration at mitochondrial complex I to induce ROS level and alter redox homoeostasis in MEFs.^31^ It was possible that mdivi-1 inhibited oxidative metabolism through increasing levels of ROS in cancer cells. Consistent with this, H_2_O_2_ and mdivi-1 decreased the levels of enriched α-KG and malate to a similar degree, and this repression was blocked by the ROS scavenger NAC in H460 cells. We also treated H460 cells with NMN and FK866 to alter the NAD+/NADH ratio, but neither treatment altered the enrichment of TCA cycle intermediates. Our data suggest that mdivi-1-mediated decreases in oxidative metabolism are independent redox alteration but associated with elevated ROS levels in the cancer cells.

Of significance in regards to results showing that mdivi-1 reduces tumour growth,^4,22^ our study revealed that inhibition of oxidative metabolism underlines the anti-proliferative effect of mdivi-1 on cancer cells. Further studies are required to evaluate how altered mitochondrial dynamics regulate metabolic reprogramming in cancer cells.

## Supplementary information

Supplemental Material

Table S1

Online supporting information

## Data Availability

The data generated and/or analysed during the current study are available from the corresponding author on reasonable request.

## References

[1] Warburg O (1925). Iron, the oxygen-carrier of respiration-ferment. Science.

[2] Otto AM (2016). Warburg effect(s)-a biographical sketch of Otto Warburg and his impacts on tumor metabolism. Cancer Metab..

[3] Jiang L, Deberardinis RJ (2012). Cancer metabolism: when more is less. Nature.

[4] Rehman J, Zhang HJ, Toth PT, Zhang Y, Marsboom G, Hong Z (2012). Inhibition of mitochondrial fission prevents cell cycle progression in lung cancer. FASEB J..

[5] Zhao J, Zhang J, Yu M, Xie Y, Huang Y, Wolff DW (2013). Mitochondrial dynamics regulates migration and invasion of breast cancer cells. Oncogene.

[6] Tanwar DK, Parker DJ, Gupta P, Spurlock B, Alvarez RD, Basu MK (2016). Crosstalk between the mitochondrial fission protein, Drp1, and the cell cycle is identified across various cancer types and can impact survival of epithelial ovarian cancer patients. Oncotarget.

[7] Cheng X, Zhou D, Wei J, Lin J (2013). Cell-cycle arrest at G2/M and proliferation inhibition by adenovirus-expressed mitofusin-2 gene in human colorectal cancer cell lines. Neoplasma.

[8] Inoue-Yamauchi A, Oda H (2012). Depletion of mitochondrial fission factor DRP1 causes increased apoptosis in human colon cancer cells. Biochem. Biophys. Res. Commun..

[9] Scheideler M, Herzig S (2017). Let’s burn whatever you have: mitofusin 2 metabolically re-wires brown adipose tissue. EMBO Rep..

[10] Kashatus JA, Nascimento A, Myers LJ, Sher A, Byrne FL, Hoehn KL (2015). Erk2 phosphorylation of Drp1 promotes mitochondrial fission and MAPK-driven tumor growth. Mol. Cell.

[11] Orang AV, Petersen J, McKinnon RA, Michael MZ (2019). Micromanaging aerobic respiration and glycolysis in cancer cells. Mol. Metab..

[12] Sellers K, Allen TD, Bousamra M2nd, Tan J, Mendez-Lucas A, Lin W (2019). Metabolic reprogramming and Notch activity distinguish between non-small cell lung cancer subtypes. Br. J. Cancer.

[13] Cairns RA, Harris IS, Mak TW (2011). Regulation of cancer cell metabolism. Nat. Rev. Cancer.

[14] DeBerardinis RJ, Thompson CB (2012). Cellular metabolism and disease: what do metabolic outliers teach us?. Cell.

[15] Lewis MR, Lewis WH (1915). Mitochondria (and other cytoplasmic structures) in tissue cultures. Am. J. Anat..

[16] Archer SL (2013). Mitochondrial dynamics-mitochondrial fission and fusion in human diseases. N. Engl. J. Med..

[17] Chan DC (2012). Fusion and fission: interlinked processes critical for mitochondrial health. Annu. Rev. Genet..

[18] Chen H, Chan DC (2017). Mitochondrial dynamics in regulating the unique phenotypes of cancer and stem cells. Cell Metab..

[19] Dai W, Jiang L (2019). Dysregulated mitochondrial dynamics and metabolism in obesity, diabetes, and cancer. Front. Endocrinol..

[20] Desmoulins L, Chretien C, Paccoud R, Collins S, Cruciani-Guglielmacci C, Galinier A (2019). Mitochondrial dynamin-related protein 1 (DRP1) translocation in response to cerebral glucose is impaired in a rat model of early alteration in hypothalamic glucose sensing. Mol. Metab..

[21] Sharp WW, Fang YH, Han M, Zhang HJ, Hong Z, Banathy A (2014). Dynamin-related protein 1 (Drp1)-mediated diastolic dysfunction in myocardial ischemia-reperfusion injury: therapeutic benefits of Drp1 inhibition to reduce mitochondrial fission. FASEB J..

[22] Ferreira-da-Silva, A., Valacca, C., Rios, E., Populo, H., Soares, P., Sobrinho-Simoes, M. et al. Mitochondrial dynamics protein Drp1 is overexpressed in oncocytic thyroid tumors and regulates cancer cell migration. *PLoS ONE* **10**, e0122308 (2015). 10.1371/journal.pone.0122308 PMC4379140 25822260

[23] Li J, Huang Q, Long X, Guo X, Sun X, Jin X (2017). Mitochondrial elongation-mediated glucose metabolism reprogramming is essential for tumour cell survival during energy stress. Oncogene.

[24] Xie Q, Wu Q, Horbinski CM, Flavahan WA, Yang K, Zhou W (2015). Mitochondrial control by DRP1 in brain tumor initiating cells. Nat. Neurosci..

[25] Peiris-Pages M, Bonuccelli G, Sotgia F, Lisanti MP (2018). Mitochondrial fission as a driver of stemness in tumor cells: mDIVI1 inhibits mitochondrial function, cell migration and cancer stem cell (CSC) signalling. Oncotarget.

[26] Kingnate C, Charoenkwan K, Kumfu S, Chattipakorn N, Chattipakorn SC (2018). Possible roles of mitochondrial dynamics and the effects of pharmacological interventions in chemoresistant ovarian cancer. EBioMedicine.

[27] Lima, A. R., Santos, L., Correia, M., Soares, P., Sobrinho-Simoes, M., Melo, M. et al. Dynamin-related protein 1 at the crossroads of cancer. *Genes* **9**, 115 (2018). 10.3390/genes9020115 PMC5852611 29466320

[28] Tang Q, Liu W, Zhang Q, Huang J, Hu C, Liu Y (2018). Dynamin-related protein 1-mediated mitochondrial fission contributes to IR-783-induced apoptosis in human breast cancer cells. J. Cell. Mol. Med..

[29] Cassidy-Stone A, Chipuk JE, Ingerman E, Song C, Yoo C, Kuwana T (2008). Chemical inhibition of the mitochondrial division dynamin reveals its role in Bax/Bak-dependent mitochondrial outer membrane permeabilization. Dev. Cell.

[30] Smith G, Gallo G (2017). To mdivi-1 or not to mdivi-1: is that the question?. Dev. Neurobiol..

[31] Bordt EA, Clerc P, Roelofs BA, Saladino AJ, Tretter L, Adam-Vizi V (2017). The putative Drp1 inhibitor mdivi-1 is a reversible mitochondrial complex I inhibitor that modulates reactive oxygen species. Dev. Cell.

[32] Koch B, Traven A (2019). Mdivi-1 and mitochondrial fission: recent insights from fungal pathogens. Curr. Genet..

[33] Koch B, Barugahare AA, Lo TL, Huang C, Schittenhelm RB, Powell DR (2018). A metabolic checkpoint for the yeast-to-hyphae developmental switch regulated by endogenous nitric oxide signaling. Cell Rep..

[34] Jiang L, Boufersaoui A, Yang C, Ko B, Rakheja D, Guevara G (2017). Quantitative metabolic flux analysis reveals an unconventional pathway of fatty acid synthesis in cancer cells deficient for the mitochondrial citrate transport protein. Metab. Eng..

[35] Jiang L, Shestov AA, Swain P, Yang C, Parker SJ, Wang QA (2016). Reductive carboxylation supports redox homeostasis during anchorage-independent growth. Nature.

[36] Ran FA, Hsu PD, Wright J, Agarwala V, Scott DA, Zhang F (2013). Genome engineering using the CRISPR-Cas9 system. Nat. Protoc..

[37] Thorsness PE, Koshland DE (1987). Inactivation of isocitrate dehydrogenase by phosphorylation is mediated by the negative charge of the phosphate. J. Biol. Chem..

[38] Alp PR, Newsholme EA, Zammit VA (1976). Activities of citrate synthase and NAD+-linked and NADP+-linked isocitrate dehydrogenase in muscle from vertebrates and invertebrates. Biochem. J..

[39] Wang J, Hansen K, Edwards R, Van Houten B, Qian W (2015). Mitochondrial division inhibitor 1 (mdivi-1) enhances death receptor-mediated apoptosis in human ovarian cancer cells. Biochem Biophys. Res. Commun..

[40] Qian W, Wang J, Roginskaya V, McDermott LA, Edwards RP, Stolz DB (2014). Novel combination of mitochondrial division inhibitor 1 (mdivi-1) and platinum agents produces synergistic pro-apoptotic effect in drug resistant tumor cells. Oncotarget.

[41] Alkan HF, Walter KE, Luengo A, Madreiter-Sokolowski CT, Stryeck S, Lau AN (2018). Cytosolic aspartate availability determines cell survival when glutamine is limiting. Cell Metab..

[42] Yeon JY, Min SH, Park HJ, Kim JW, Lee YH, Park SY (2015). Mdivi-1, mitochondrial fission inhibitor, impairs developmental competence and mitochondrial function of embryos and cells in pigs. J. Reprod. Dev..

[43] DeBerardinis RJ, Mancuso A, Daikhin E, Nissim I, Yudkoff M, Wehrli S (2007). Beyond aerobic glycolysis: transformed cells can engage in glutamine metabolism that exceeds the requirement for protein and nucleotide synthesis. Proc. Natl Acad. Sci. USA.

[44] Guzzo G, Sciacovelli M, Bernardi P, Rasola A (2014). Inhibition of succinate dehydrogenase by the mitochondrial chaperone TRAP1 has anti-oxidant and anti-apoptotic effects on tumor cells. Oncotarget.

[45] Tretter L, Adam-Vizi V (1999). Inhibition of alpha-ketoglutarate dehydrogenase due to H2O2-induced oxidative stress in nerve terminals. Ann. NY Acad. Sci..

[46] Mailloux RJ, Jin X, Willmore WG (2014). Redox regulation of mitochondrial function with emphasis on cysteine oxidation reactions. Redox Biol..

[47] Berger F, Lau C, Dahlmann M, Ziegler M (2005). Subcellular compartmentation and differential catalytic properties of the three human nicotinamide mononucleotide adenylyltransferase isoforms. J. Biol. Chem..

[48] Di Stefano M, Nascimento-Ferreira I, Orsomando G, Mori V, Gilley J, Brown R (2015). A rise in NAD precursor nicotinamide mononucleotide (NMN) after injury promotes axon degeneration. Cell Death Differ..

[49] Zeng L, Morinibu A, Kobayashi M, Zhu Y, Wang X, Goto Y (2015). Aberrant IDH3alpha expression promotes malignant tumor growth by inducing HIF-1-mediated metabolic reprogramming and angiogenesis. Oncogene.

[50] Zhang D, Wang Y, Shi Z, Liu J, Sun P, Hou X (2015). Metabolic reprogramming of cancer-associated fibroblasts by IDH3alpha downregulation. Cell Rep..

[51] Anderson GR, Wardell SE, Cakir M, Yip C, Ahn YR, Ali M (2018). Dysregulation of mitochondrial dynamics proteins are a targetable feature of human tumors. Nat. Commun..

[52] Aouacheria A, Baghdiguian S, Lamb HM, Huska JD, Pineda FJ, Hardwick JM (2017). Connecting mitochondrial dynamics and life-or-death events via Bcl-2 family proteins. Neurochem. Int..

[53] Morciano G, Pedriali G, Sbano L, Iannitti T, Giorgi C, Pinton P (2016). Intersection of mitochondrial fission and fusion machinery with apoptotic pathways: role of Mcl-1. Biol. Cell.

[54] Sheridan C, Delivani P, Cullen SP, Martin SJ (2008). Bax- or Bak-induced mitochondrial fission can be uncoupled from cytochrome C release. Mol. Cell.

[55] Parra V, Bravo-Sagua R, Norambuena-Soto I, Hernandez-Fuentes CP, Gomez-Contreras AG, Verdejo HE (2017). Inhibition of mitochondrial fission prevents hypoxia-induced metabolic shift and cellular proliferation of pulmonary arterial smooth muscle cells. Biochim. Biophys. Acta Mol. Basis Dis..

[56] Galloway CA, Lee H, Nejjar S, Jhun BS, Yu T, Hsu W (2012). Transgenic control of mitochondrial fission induces mitochondrial uncoupling and relieves diabetic oxidative stress. Diabetes.

[57] Westermann B (2012). Bioenergetic role of mitochondrial fusion and fission. Biochim. Biophys. Acta.

[58] Manczak M, Kandimalla R, Yin X, Reddy PH (2019). Mitochondrial division inhibitor 1 reduces dynamin-related protein 1 and mitochondrial fission activity. Hum. Mol. Genet..

[59] Hong Z, Kutty S, Toth PT, Marsboom G, Hammel JM, Chamberlain C (2013). Role of dynamin-related protein 1 (Drp1)-mediated mitochondrial fission in oxygen sensing and constriction of the ductus arteriosus. Circ. Res..

[60] Cheng CT, Kuo CY, Ouyang C, Li CF, Chung Y, Chan DC (2016). Metabolic stress-induced phosphorylation of KAP1 Ser473 blocks mitochondrial fusion in breast cancer cells. Cancer Res..

[61] Dal Yontem, F., Kim, S. H., Ding, Z., Grimm, E., Ekmekcioglu, S., Akcakaya, H. Mitochondrial dynamic alterations regulate melanoma cell progression. *J. Cell. Biochem*. **120**, 2098–2108 (2019). 10.1002/jcb.27518 30256441

[62] Yu, M., Nguyen, N. D., Huang, Y., Lin, D., Fujimoto, T. N., Molkentine, J. M. et al. Mitochondrial fusion exploits a therapeutic vulnerability of pancreatic cancer. *JCI Insight*10.1172/jci.insight.126915 (2019). 10.1172/jci.insight.126915 PMC6777817 31335325

