# Supplementary material for: Mitochondrial division inhibitor (mdivi-1) decreases oxidative metabolism in cancer
Source: Br J Cancer. 2020 Mar 9;122(9):1288–97. doi: 10.1038/s41416-020-0778-x (PMC7188673; doi:10.1038/s41416-020-0778-x)

**Supplemental Information**

**Mitochondrial division inhibitor represses oxidative metabolism in cancer**

**Running title:** Mdivi-1-repressed oxidation is independent of DRP1

Wenting Dai^1^, Guan Wang^1^, Jason Chwa^1^, Myung Eun Oh^1^, Tharindumala Abeywardana^2^, Yanzhong Yang^2^, Qiong A. Wang^1,3^, Lei Jiang^1,3^*

^1^Department of Molecular and Cellular Endocrinology, Diabetes and Metabolism Research Institutes, ^2^Departments of Cancer Genetics and Epigenetics, Beckman Research Institute, ^3^Comprehensive Cancer Center, City of Hope Medical Center, Duarte, CA, USA 91010

*Correspondence: Lei Jiang ([ljiang@coh.org](mailto:ljiang@coh.org)); Phone: 1-626-218-6401.

1500 E. Duarte Road, Duarte, CA, USA 91010-3000

**Table S1** **The relative levels of metabolites in three cancer cell lines (affiliated to Fig. 2).**

|  | H460 | | | A549 | | | HCT116 | | |
| --- | --- | --- | --- | --- | --- | --- | --- | --- | --- |
|  | DMSO | 50uM mdivi-1 | 50uM mdivi-1 Pre-treatment | DMSO | 50uM mdivi-1 | 50uM mdivi-1 Pre-treatment | DMSO | 50uM mdivi-1 | 50uM mdivi-1 Pre-treatment |
| Cysteine | 1±0.11 | 1.33±0.04* | 1.25±0.13 | 1±0.31 | 0.85±0.29 | 0.82±0.16 | 1±0.19 | 0.75±0.09 | 0.63±0.02* |
| DHAP | 1±0.34 | 1.17±0.49 | 0.51±0.04 | 1±0.09 | 1.00±0.01 | 1.29±0.23* | 1±0.03 | 0.64±0.05* | 0.90±0.28* |
| Glutamine | 1±0.04 | 1.17±0.12 | 1.07±0.16 | 1±0.07 | 0.96±0.02 | 0.98±0.06 | 1±0.01 | 0.91±0.03* | 1.11±0.02* |
| 3-PG | 1±0.11 | 1.10±0.03 | 1.06±0.15 | 1±0.13 | 1.16±0.14 | 0.62±0.06* | 1±0.31 | 0.82±0.23 | 1.02±0.22 |
| Isoleucine | 1±0.02 | 1.09±0.01* | 1.00±0.07 | 1±0.06 | 1.02±0.03 | 0.98±0.05 | 1±0.01 | 1.09±0.03* | 1.35±0.05* |
| Tyrosine | 1±0.01 | 1.08±0.02 | 0.95±0.07 | 1±0.06 | 1.08±0.07 | 1.08±0.02 | 1±0.02 | 1.12±0.02* | 1.35±0.03* |
| Proline | 1±0.05 | 1.07±0.03 | 1.12±0.09 | 1±0.09 | 0.96±0.06 | 0.91±0.07 | 1±0.48 | 1.19±0.13 | 1.44±0.35 |
| GAP | 1±0.15 | 1.05±0.24 | 0.68±0.07 | 1±0.09 | 1.04±0.01 | 1.29±0.19* | 1±0.08 | 0.24±0.02* | 0.33±0.05* |
| Leucine | 1±0.03 | 1.04±0.02 | 0.90±0.06* | 1±0.06 | 1.02±0.03 | 0.99±0.06 | 1±0.02 | 1.09±0.02* | 1.35±0.06* |
| Pyruvate | 1±0.39 | 1.03±0.19 | 1.06±0.03 | 1±0.09 | 1.42±0.04* | 1.58±0.09* | 1±0.08 | 1.30±0.16* | 1.39±0.15* |
| Glycine | 1±0.02 | 1.03±0.02 | 1.05±0.09 | 1±0.06 | 0.98±0.03 | 0.93±0.07 | 1±0.01 | 0.99±0.01 | 1.19±0.03* |
| Lactate | 1±0.02 | 1.02±0.09 | 0.99±0.09 | 1±0.09 | 1.26±0.06* | 1.10±0.07 | 1±0.02 | 1.07±0.02 | 1.24±0.08* |
| Phenylalanine | 1±0.02 | 1.00±0.04 | 0.84±0.07* | 1±0.06 | 1.08±0.04 | 1.04±0.08 | 1±0.04 | 1.17±0.02* | 1.47±0.07* |
| Methionine | 1±0.03 | 0.98±0.04 | 0.85±0.06* | 1±0.06 | 1.06±0.02 | 1.00±0.04 | 1±0.03 | 1.12±0.02* | 1.30±0.07* |
| Histidine | 1±0.07 | 0.97±0.02 | 1.01±0.03 | 1±0.08 | 1.12±0.11 | 1.13±0.06 | 1±0.06 | 1.26±0.07* | 1.68±0.07* |
| Valine | 1±0.02 | 0.97±0.03 | 0.83±0.05* | 1±0.06 | 1.01±0.03 | 0.98±0.06 | 1±0.02 | 1.09±0.03* | 1.39±0.07* |
| Threonine | 1±0.03 | 0.95±0.02 | 0.94±0.05 | 1±0.08 | 0.99±0.09 | 0.97±0.09 | 1±0.04 | 0.98±0.03 | 1.2±0.03* |
| Glutamate | 1±0.03 | 0.95±0.02 | 0.94±0.04 | 1±0.05 | 1.00±0.01 | 0.92±0.06 | 1±0.02 | 0.94±0.02* | 1.06±0.03* |
| α-KG | 1±0.04 | 0.93±0.01 | 0.73±0.04* | 1±0.11 | 0.93±0.08 | 0.75±0.11 | 1±0.23 | 1.32±0.11 | 1.28±0.01 |
| Serine | 1±0.03 | 0.91±0.03* | 0.83±0.05* | 1±0.06 | 0.99±0.04 | 0.93±0.05 | 1±0.02 | 0.97±0.01 | 1.26±0.04* |
| Alanine | 1±0.02 | 0.91±0.08 | 0.75±0.05* | 1±0.08 | 0.99±0.02 | 0.98±0.08 | 1±0.04 | 0.98±0.03 | 0.84±0.04* |
| Fumarate | 1±0.01 | 0.83±0.05* | 0.58±0.06* | 1±0.05 | 1.18±0.13* | 0.73±0.06* | 1±0.03 | 0.76±0.05* | 0.73±0.05* |
| Aspartate | 1±0.02 | 0.64±0.01* | 0.43±0.04* | 1±0.05 | 0.86±0.03* | 0.73±0.09* | 1±0.06 | 0.47±0.01* | 0.41±0.00* |
| Citrate | 1±0.13 | 0.64±0.05* | 0.53±0.08* | 1±0.09 | 0.32±0.07* | 0.39±0.06* | 1±0.03 | 0.28±0.01* | 0.18±0.01* |
| Malate | 1±0.09 | 0.62±0.07* | 0.46±0.01* | 1±0.06 | 0.80±0.02* | 0.66±0.06* | 1±0.05 | 0.64±0.01* | 0.55±0.05* |

In the same experiment as showed in **Fig. 2**, H460, A549, and HCT116 cancer cells were pre-treated with or without 50 μM mdivi-1 for 6 hours and then cultured in medium containing [U-^13^C]glucose for 2 hours. G3P, glyceraldehyde 3-phosphate; DHAP, dihydroxyacetone phosphate; 3-PG, 3-phosphoglycerate; α-KG, alpha-ketoglutarate. Data are represented as mean ± S.D (n=3). The data were analyzed by one-way ANOVA followed by Tukey's multiple comparisons test, and the significant level was set as *p < 0.05, comparing to DMSO control.

**Figure S1**





**Fig. S1 Chronic mdivi-1 treatment inhibited oxidative metabolism in H460 cells** (affiliated to **Fig. 1**). H460 lung cancer cells were treated with DMSO (control) and 20 μM mdivi-1 in medium containing [U-^13^C]glucose for 8 hours (**a** and **c**) and 24 hours (**b, d** and **e**). **a, b** The metabolic enrichment of glycolytic intermediates are shown as m+3 pyruvate and lactate. **c, d** The metabolic enrichment of TCA cycle intermediates are shown as m+2 citrate, α-ketoglutarate (α-KG), fumarate, and malate. **e** After multiple rounds of TCA cycle, the metabolic enrichment of α-KG are shown as m+3 and m+4 in H460 cells culture with [U-^13^C]glucose for 24 hours. Data are represented as mean ± S.D (n=3). The data were analyzed by Student’s t-test, and the significant level was set as **p* < 0.05, comparing to DMSO control.

**Figure S2**





**Fig. S2 50 μM mdivi-1 treatment decreased the abundance of citrate and malate in multiple cancer cell lines** (affiliated to **Fig. 2**). In the same experiment as showed in Fig. 2, H460, A549, and HCT116 cancer cells were pre-treated with or without 50 μM mdivi-1 for 6 hours and then cultured in medium containing [U-^13^C]glucose for 2 hours. The relative levels of citrate (**a**), α-ketoglutarate (α-KG) (**b**), and malate (**c**) in all three cancer cell lines. Data are represented as mean ± S.D (n=3). The data were analyzed by one-way ANOVA followed by Tukey's multiple comparisons test, and the significant level was set as **p* < 0.05, comparing to DMSO control.

**Figure S3**





**Fig. S3 Long time mdivi-1 treatment repressed oxidative metabolism in multiple cell lines.** H460, A549, and HCT116 cancer cells were pre-treated with or without 20 μM mdivi-1 for 6 hours and then cultured in medium containing [U-^13^C]glucose for 2 hours. The metabolic enrichment of TCA cycle intermediates are shown as m+2 citrate (**a**), α-ketoglutarate (α-KG) (**b**), and malate (**c**) in all three cancer cell lines. Data are represented as mean ± S.D (n=3). The data were analyzed by one-way ANOVA followed by Tukey's multiple comparisons test, and the significant level was set as **p* < 0.05, comparing to DMSO control.

**Figure S4**





**Fig. S4** **DRP1 deficient MEFs had normal oxidative metabolism**. **a-c** Drp1-WT and Drp1-KO MEFs were cultured in medium containing [U-^13^C]glucose for 4 hours. The metabolic enrichment of TCA cycle intermediates are shown as citrate (**a**), α-ketoglutarate (α-KG) (**b**), and malate (**c**). **d** The metabolic enrichment of m+2 α-KG in Drp1-WT and Drp1-KO MEFs pre-treated with or without 50 μM mdivi-1 for 6 hours and cultured in medium containing [U-^13^C]glucose for 4 hours. Data are represented as mean ± S.D (n=3). The data in **a-c** were analyzed by Student’s t-test, and the significant level was set as * *p* < 0.05, comparing to Drp1-WT. The data in **d** were analyzed by two-way ANOVA followed by Tukey's multiple comparisons test, and the significant level was set as * *p* < 0.05 (50 μM mdivi-1 vs. DMSO).

**Figure S5**

**
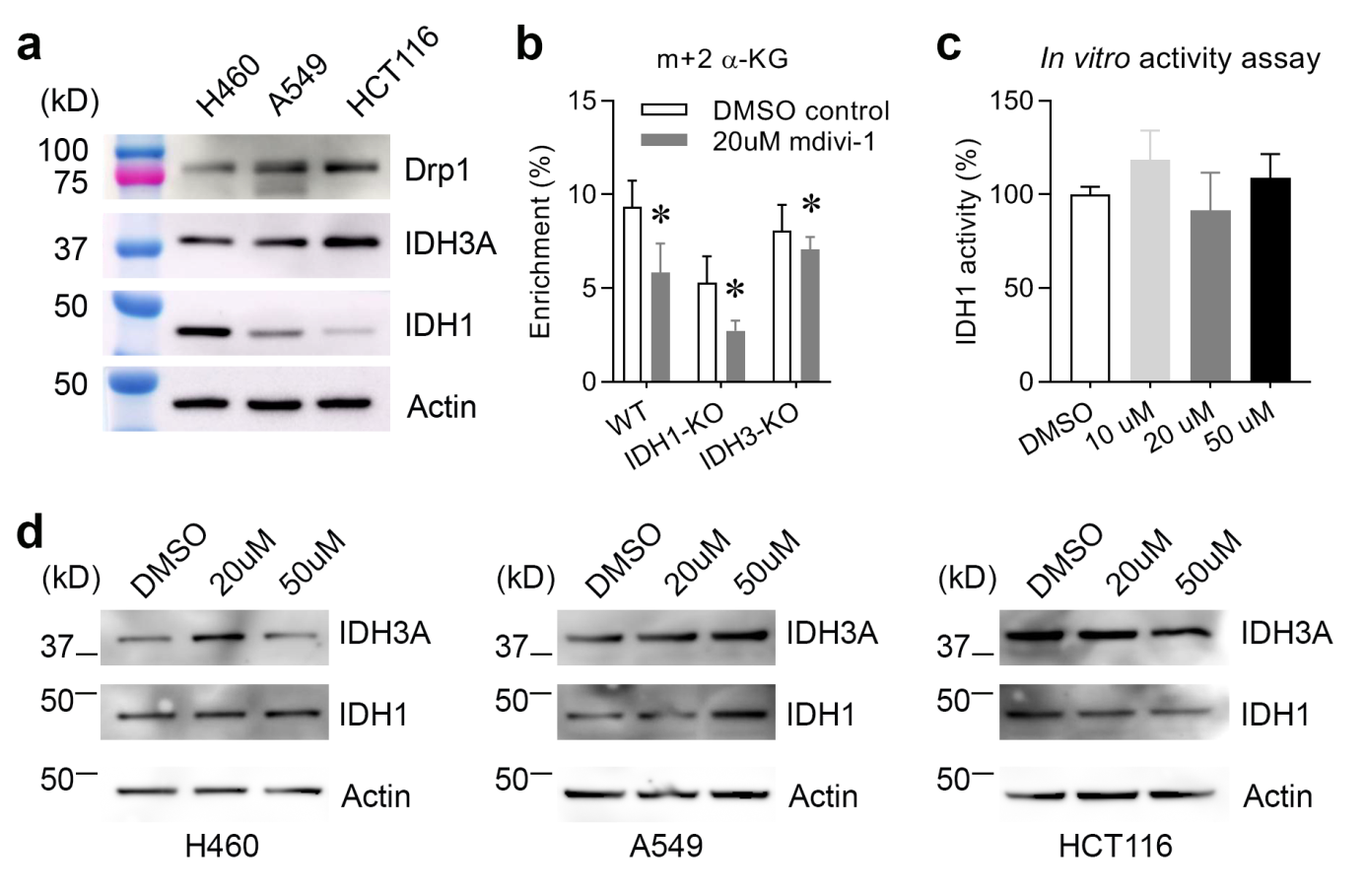
**

**Fig. S5 Mdivi-1 repressed IDH1/3-mediated oxidative α-KG production. a** Immunoblotting of DRP1, IDH1 and IDH3A proteins in H460, A549, and HCT116 cancer cell lines. **b** The metabolic enrichment of m+2 α-ketoglutarate (α-KG) in WT, IDH1-KO and IDH3-KO H460 cells treated with DMSO and 20 μM mdivi-1 for 6 hours, and then cultured in medium containing [U-^13^C]glucose for 2 hours. **c** *In vitro* enzyme activity assay of purified IDH1 protein treated with mdivi-1. **d** Immunoblotting of IDH1 and IDH3A proteins in H460, A549, and HCT116 cells treated with DMSO, 20 μM and 50 μM mdivi-1 for 8 hours. Data are represented as mean ± S.D (n=3). The data in **b** were analyzed by two-way ANOVA followed by Tukey's multiple comparisons test, and the significant level was set as * *p* < 0.05, comparing to DMSO control. The data in **c** were analyzed by one-way ANOVA followed by Tukey's multiple comparisons test.

**Figure S6**


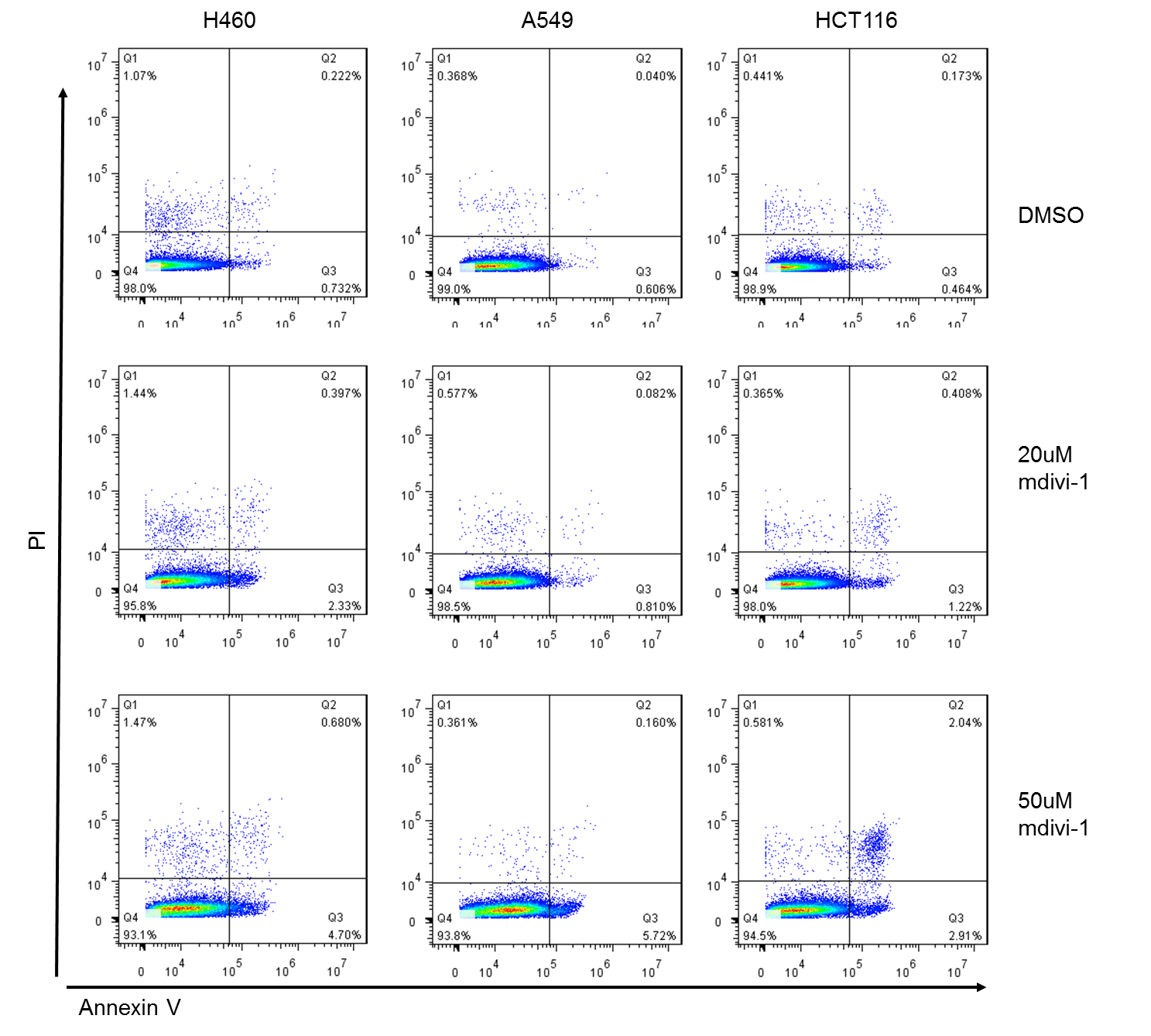


**Fig. S6 Quantitative analysis of apoptotic H460, A549 and HCT116 cells by annexin V and PI flow cytometry.** Upper right quadrant: late-stage apoptotic cells; upper left quadrant: necrotic cells; lower left quadrant: normal viable cells; lower right quadrant: early stage apoptotic cells.

**Figure S7, Full enrichment of TCA metabolites.**

**Fig 1**


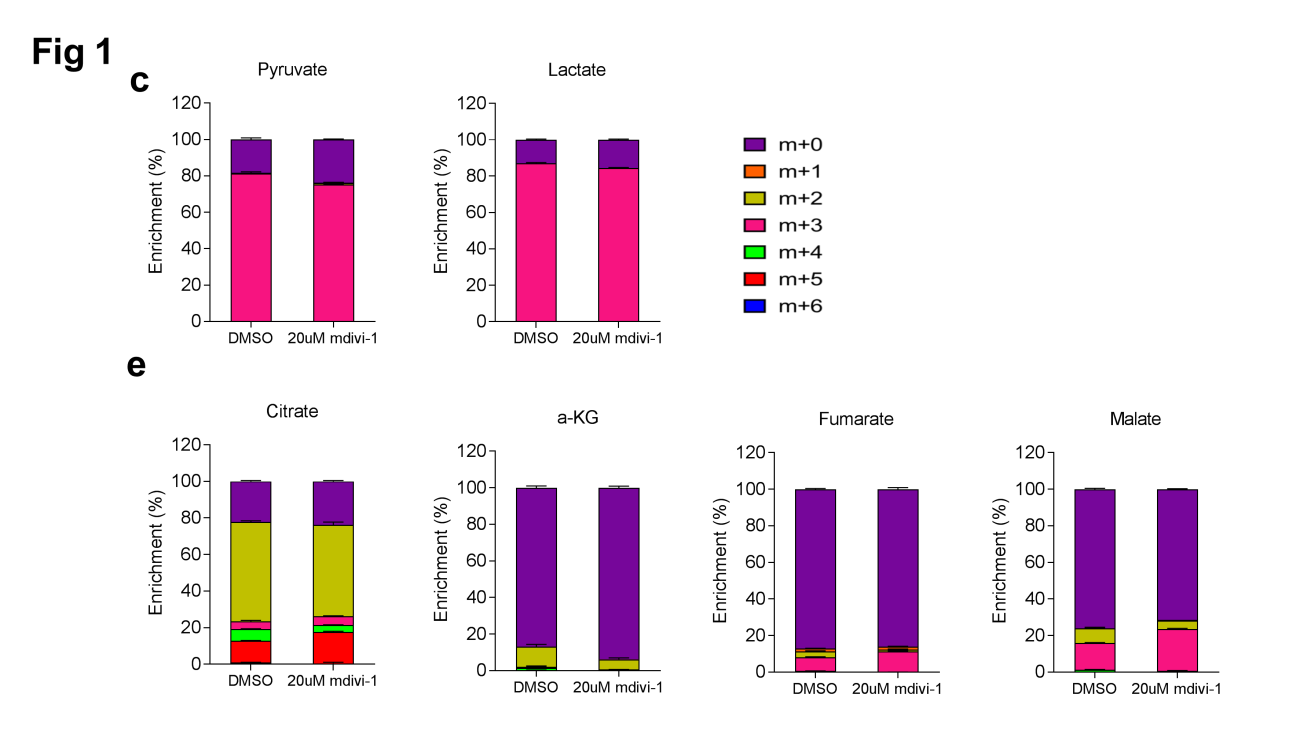


**
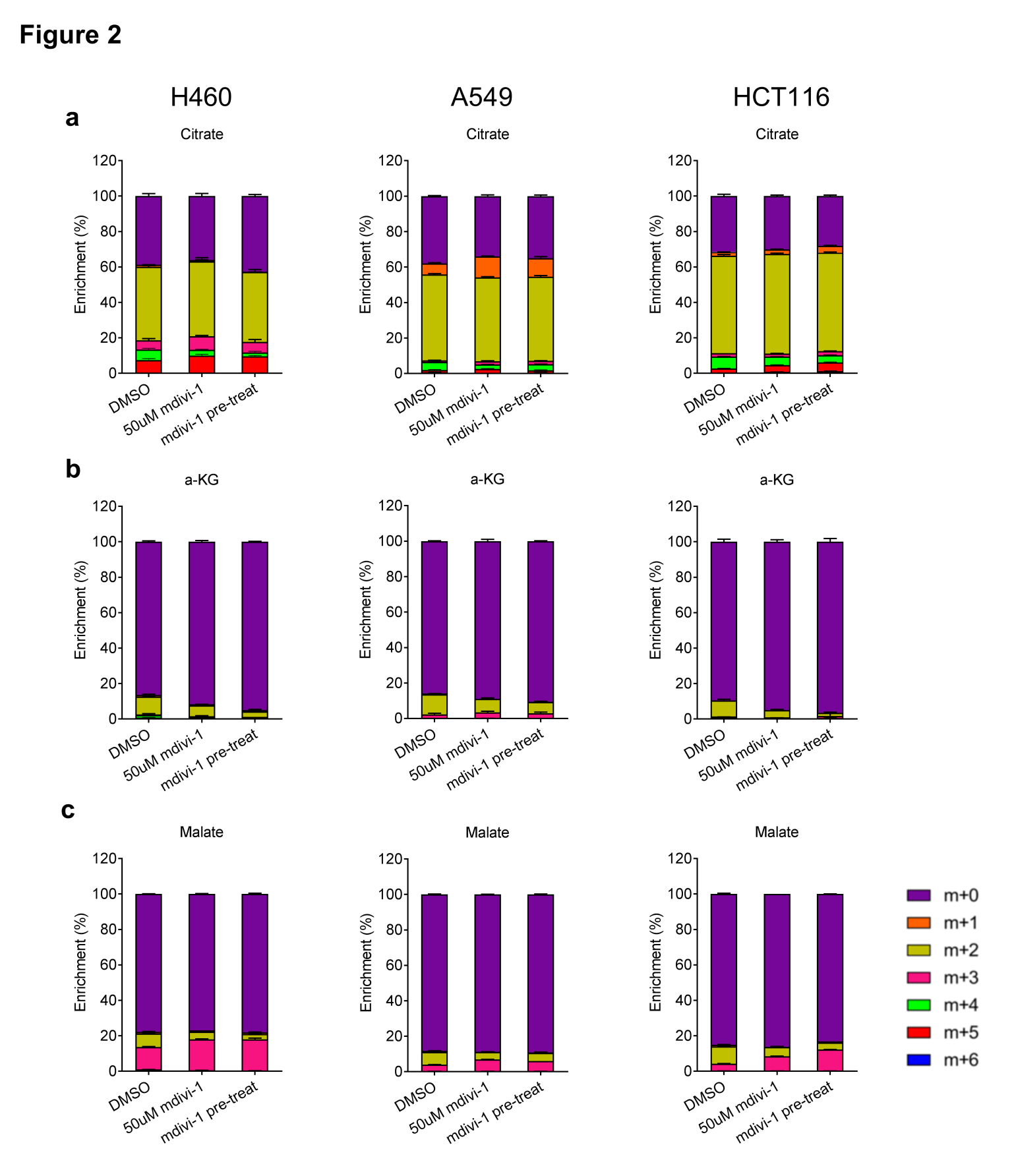
Fig 2**

**Figure S7, Full enrichment of TCA metabolites. (Continue)**


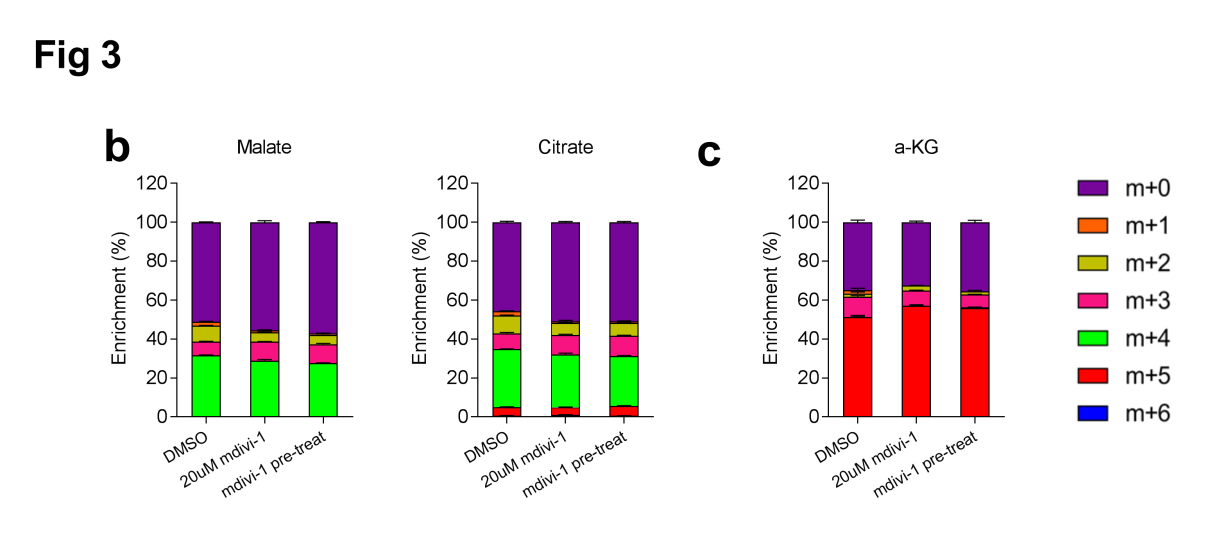


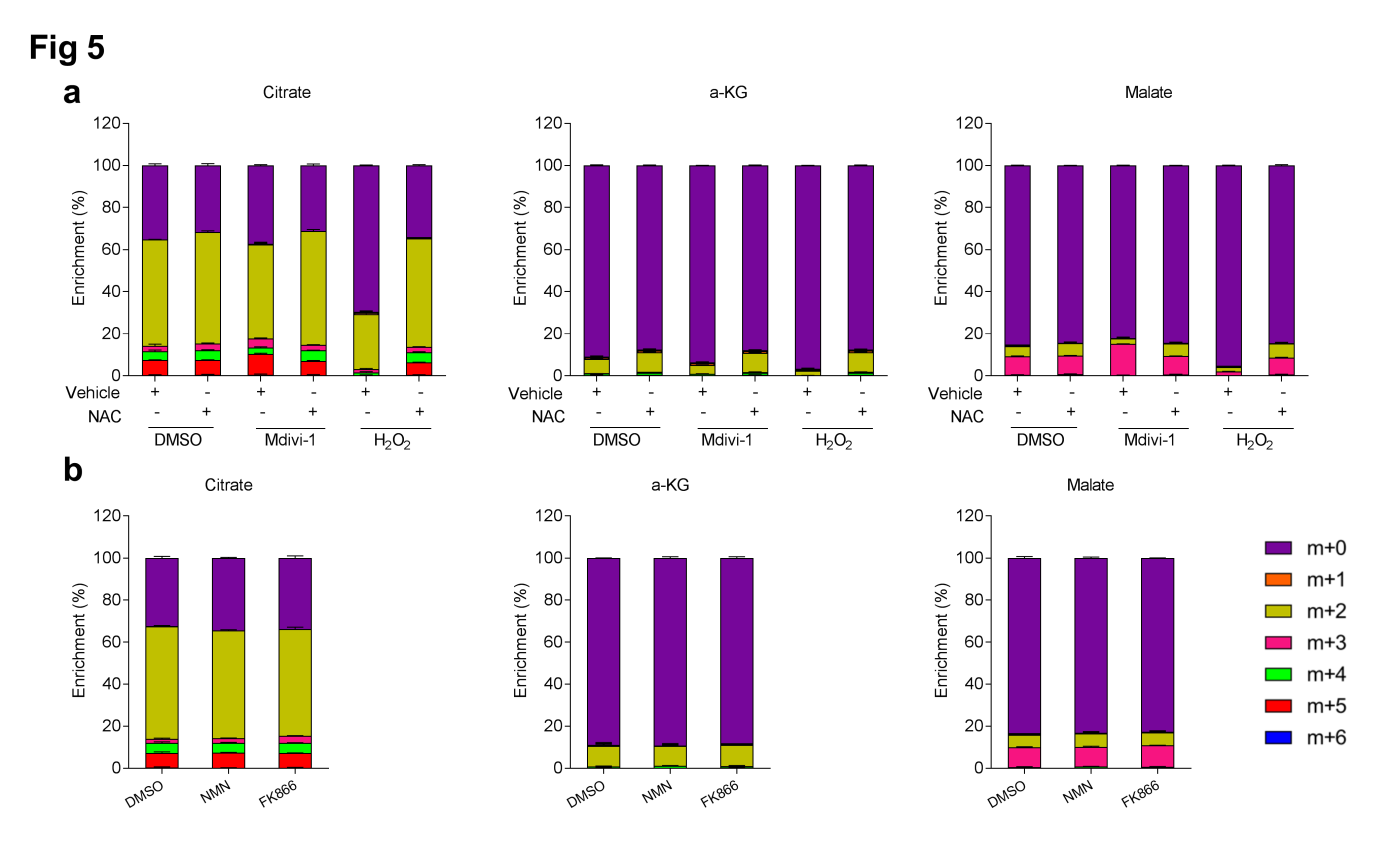


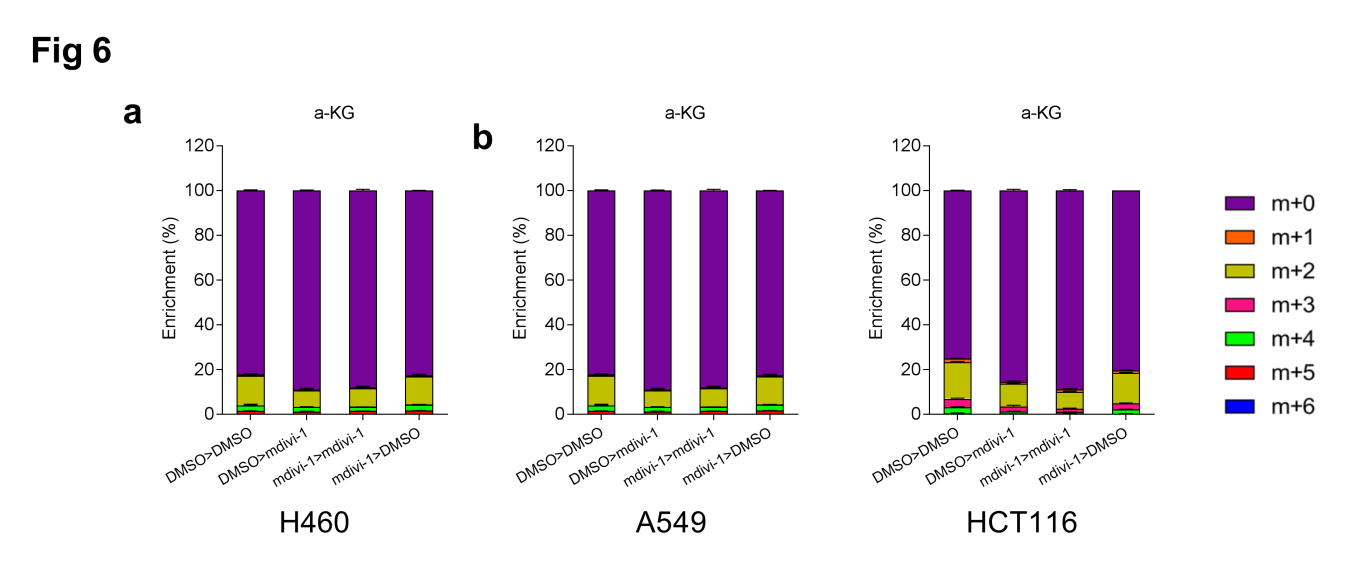

Supplement: Supplementary file 1 — Supplemental Material [file 41416_2020_778_MOESM1_ESM.docx]
